# EVALUATING THE STAFFING METHODOLOGY MODEL IN THE VETERANS HEALTH ADMINISTRATION’S COMMUNITY LIVING CENTERS

**DOI:** 10.1093/geroni/igac059.2157

**Published:** 2022-12-20

**Authors:** Lana Brown, Shelly Lensing, Sheila Cox Sullivan, Teresa Odom, Jade Moore, Pamela Billings, Lisa Minor

**Affiliations:** Central Arkansas Veterans Healthcare System, North Little Rock, Arkansas, United States; Central Arkansas for Veterans Healthcare System, North Little Rock, Arkansas, United States; Veterans Health Administration, Washington, District of Columbia, United States; Veterans Health Administration, Washington, District of Columbia, United States; Veterans Health Administration, Washington, District of Columbia, United States; Veterans Health Administration, Nashville, Tennessee, United States; Veterans Health Administration, Washington, District of Columbia, United States

## Abstract

The project purpose was to describe nursing hours per patient day (NHPPD) within the Veterans Health Administration (VHA) and to evaluate Staffing Methodology in the VHA Community Living Centers (CLCs) by comparing Staffing Methodology targeted NHPPD with actual NHPPD. Data were compiled retrospectively for each CLC unit over a one-year timeframe for calendar year 2019. VHA had 134 CLCs including 390 nursing units. Nursing units without 6 months of actual NHPPD data were excluded from the data set. For descriptive analyses, actual NHPPD were averaged across months for each unit. The percent of target was calculated as this average divided by the NHPPD target times 100. The percentage of months each unit met the targeted NHPPD was calculated. After data exclusions, the final data set included 322 nursing units from 133 CLCs. The mean for actual hours as a percent of target was 121.6% (95% CI, 118.5 to 124.7%) indicating that the units’ average hours across 2019 were 21.6% significantly higher than target. Also, half of units met their targets 91.7% of months as reflected by the median. The actual NHPPD significantly differed across months (p< 0.001) with the 2019 months of January and October having the highest NHPPD. Similar results were seen for NHPPD as a percent of target. Veteran safety is a VHA priority and appropriate nurse staffing is key to providing care that improves Veteran outcomes and reduces adverse events. Further exploration is needed on how nurse staffing in the CLCs impacts Veteran outcomes, safety, and satisfaction.

